# Evolution’s cartographer: mapping the fitness landscape in cancer

**DOI:** 10.1016/j.ccell.2021.09.002

**Published:** 2021-09-30

**Authors:** Calum Gabbutt, Trevor A Graham

**Affiliations:** 1 Centre for Genomics and Computational Biology, Barts Cancer Institute, Barts and the London School of Medicine and Dentistry, Queen Mary University of London, Charterhouse Sq, London, EC1M 6BQ

## Abstract

Cancer treatment effectiveness could be improved if it were possible to accurately anticipate the response of the tumour to treatment. Writing in Nature, Salehi and colleagues combine single-cell genomics and mathematical modelling to measure cancer subclone fitness and use these measurements to accurately predict the future trajectory of cancer evolution.

Clinical management of cancer is centred upon trying to predict the future. If we predict that a tumour isn’t too aggressive we might decide that systemic treatment and the associated risk of side effects isn’t justified. If we can predict evolution induced by treatment, we can hope to be ready with the next treatment line designed to exploit newly evolved vulnerabilities. But how predictable is cancer?

There is plenty of reason to think that cancer is a predictable disease. The current mainstay of clinical prognostication is pathological staging, and tumours of the same type and grade tend to have similar outcomes across patients. A century of clinical experience has taught us that we can predict the future of a cancer based upon our experience of similar tumours we’ve observed in the past. Recent applications of artificial intelligence to analyse pathology slides shows how subtle morphological features of cancer cells can accurately predict disease course^1^.

Mechanistically, cancer is driven by evolution. A tumour cell may acquire a new heritable alteration that increases its likelihood of producing surviving offspring – meaning that this cell has increased its *fitness* – which leads to a clonal expansion. Fundamentally then, predicting cancer requires predicting the course of an evolutionary process^2^. The success of pathological staging shows us that we can predict general properties of the evolutionary process (e.g. average time to lethal disease) but an open question is whether or not it is possible to predict the *specific* properties of the evolutionary process. If a tumour today consists of three clones, A, B and C in given proportions (Figure), can we predict what clonal make-up will be in a few months’ time, or after treatment exposure?

Forecasting how a tumour will evolve is likely to be clinically important, as the dominance or scarcity of a particular clone might guide our treatment choice. Indeed, tumour evolution in response to treatment provides the intellectual underpinning for *adaptive therapy*, where dosing is modulated in accordance with the tumour’s response to the previous line of treatment, with the intention of *steering* evolution in a direction that keeps the tumour susceptible to treatment for maximal time^3^.

To predict clonal expansions requires measurement of the relative *fitness* of each clone: fitter clones will expand and less-fit clones will contract within the tumour (Figure). Accordingly, evolutionary biology has long been concerned with measurement of the so-called “fitness landscape”^4^ – the relationship between a cell’s genotype and fitness. Researchers have begun mapping the fitness landscape in cancer^5,6^, but efforts are particularly hindered by a lack of high-resolution serial measurements.

Salehi and colleagues, in their recent paper in *Nature*^7^, tackled the interrelated problems of measuring clone fitness and predicting cancer evolution. They developed *in vitro* and murine patient derived xenograft (PDX) breast cancer models where tumour cells could be longitudinally sampled through the course of their evolution and employed single-cell genome sequencing to measure the clonal composition at serial time points. Then, using computational methods that implemented populations genetics mathematical theory, they quantified clone fitness from the observed clone frequencies, and were able to accurately predict the future evolutionary trajectory of the clones.

The paper reports multiple technological advances: in single-cell genomics for very high-throughput genomic profiling, in bioinformatics rising to the challenges involved analysing the genomes of 1,000s of cells simultaneously, and in applying population genetics theory to cancer genome data.

The authors previously established “direct library preparation” methodology was used to detect copy number alterations (CNAs; loss and gain of large-sections of genetic material) in individual cells. The measurements were performed at tremendous scale: 42,000 individual cell genomes were generated in total. To identify clones, the authors needed to infer the ancestral relationships between each cell. Processing such a large set of single-cell genomes presents significant computational challenges, which the authors tackled by creating a custom phylogenetic tree reconstruction tool called *sitka*. S*itka* introduces an innovative “two-pass” tree reconstruction approach, which first identifies major clones before filling in the detail of the phylogenetic relationships between individual cells. Clone abundance is quantified by the number of cells in each clade of the phylogenetic tree.

To measure clone fitness, Salehi *et al.* turned to population genetics mathematical theory. The Wright-Fisher mathematical model describes the expansion and contraction of competing lineages over time^8^. Using sophisticated statistical inference methods Salehi fitted the Wright-Fisher model to the observed clone frequencies – and importantly also changes in clone frequencies over time – and were able to infer the fitness of each individual subclone. These values could then be plugged back into the Wright-Fisher model to make predictions about the future dynamics.

Loss-of-function mutations in TP53, the important tumour suppressor gene, were observed to significantly increase clone fitness in an immortalised normal breast epithelial cell-line. p53-proficient cells were largely diploid and exhibited only minor differences in selection coefficients between lineages, whereas p53-deficient clones became aneuploid and in some cases genome doubled. The largest increases in fitness were observed in p53-deficient clones that had also evolved amplifications at known breast cancer driver genes. It appeared that TP53 loss-of-function shifts and broadens the fitness landscape, increasing the fitness of CNAs that were previously unfavourable and potentially also increasing the CNA mutation rate.

In untreated PDXs, fierce competition between distinctly aneuploid subclones was measured, adding to the weight of evidence that CNAs are “functional” and determine fitness in cancer evolution^9^. PDXs were also used to evaluate forecasts of evolutionary dynamics: the authors measured the fitness of individual clones, then constructed new transplants of the clones mixed in known proportions. In all cases, the empirically observed evolutionary trajectories were consistent with the predictions of the mathematical forecasts, with the clone measured to have the highest fitness in the initial mixture found to have expanded the most in the final tumour.

The same PDX models were also used to study the evolution induced by platinum-based chemotherapy (cisplatin). Following treatment, newly dominant clones had distinct CNAs from untreated cells, indicating that chemotherapy changed the fitness landscape. Interestingly, when the evolution was allowed to continue in the absence of treatment, the residual pre-treatment clones in the PDX re-expanded to the detriment of the emerged post-treatment clones. This suggested that resistance to platinum-based chemotherapy carries a fitness cost, meaning that drug-resistant cells could potentially be removed from a tumour simply by prescribing a treatment-holiday that allows the drug-sensitive cells to outcompete their rivals. Salehi and colleagues’ data is a provocative suggestion that an adaptive regimen^3^ could be efficacious for platinum therapy in breast cancer.

Salehi and colleagues’ work is an elegant *tour de force* which, importantly and excitingly, demonstrates that quantitative and accurate prediction about the dynamics of tumour evolution is possible. Going forward, it will also be important to combine a “clonal dynamics” forecast with a prediction about the likelihood that new previously undetected clones will arise in a tumour, especially in the setting of mutagenic chemotherapies^10^. The newly constructed map of cancer evolution enables us to see where a cancer is headed, offering the tantalising possibility that we can design our treatments to keep one step ahead of a tumour’s evolutionary journey.

## Figures and Tables

**Figure:**
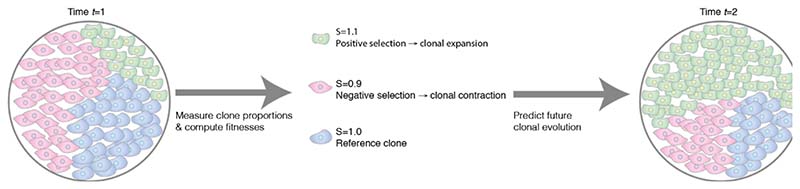
Measuring clonal composition and inferring fitness to predict cancer evolution. Salehi and colleagues used single-cell genome sequencing to measure the size of subclones within cell cultures and PDX models. Clone sizes were then inputted into a population genetics mathematical model to infer clone-specific fitness coefficients. The same mathematical model could then be run-forwards in time to make predictions about future evolution.
